# Combination phase I study of nedaplatin and gemcitabine for advanced non-small-cell lung cancer

**DOI:** 10.1038/sj.bjc.6601817

**Published:** 2004-04-20

**Authors:** T Kurata, K Tamura, N Yamamoto, T Nogami, T Satoh, H Kaneda, K Nakagawa, M Fukuoka

**Affiliations:** 1 Department of Medical Oncology, Kinki University School of Medicine: 377-2, Ohno-Higashi, Osaka-Sayama, Osaka 589-8511, Japan

**Keywords:** combination phase I study, maximum tolerated dose, nedaplatin, gemcitabine, non-small-cell lung cancer

## Abstract

To establish the toxicities and maximum tolerated dose (MTD) of nedaplatin with gemcitabine, and to observe their antitumour activity, we conducted a combination phase I study in advanced non-small-cell lung cancer (NSCLC). Patients received nedaplatin (60–100 mg m^−2^ given intravenously over 90 min) on day 1, and gemcitabine (800–1000 mg m^−2^ given intravenously over 30 min) on days 1, 8, every 3 weeks. In total, 20 patients with locally advanced or metastatic NSCLC who received no prior chemotherapy or one previous chemotherapy regimen were enrolled. The most frequent toxicities were neutropenia and thrombocytopenia; nonhaematological toxicities were generally mild. Three out of six patients experienced dose-limiting toxicities (neutropenia, thrombocytopenia and delayed anaemia) at dose level 4, 100 mg m^−2^ nedaplatin with 1000 mg m^−2^ gemcitabine, which was regarded as the MTD. There were three partial responses, for an overall response rate of 16.7%. The median survival time and 1-year survival rate were 9.1 months and 34.1%, respectively. This combination is well tolerated and active for advanced NSCLC. The recommended dose is 80 mg m^−2^ nedaplatin with 1000 mg m^−2^ gemcitabine. This combination chemotherapy warrants a phase II study and further evaluation in prospective randomised trials with cisplatin- or carboplatin-based combinations as first-line chemotherapy for advanced NSCLC.

Based on the results of a meta-analysis (Non-Small Cell Lung Cancer Collaborative Group, 1995), cisplatin-based chemotherapy is considered the best available therapy for patients with locally advanced or metastatic non-small-cell lung cancer (NSCLC). Although several new agents with novel mechanisms and significant activity against NSCLC have been introduced, such as taxanes, gemcitabine and vinorelbine, any of these agents used in combination with a platinum agent provide equivalent survival improvement (Kelly *et al*, 2001; Schiller *et al*, 2002; Fossella *et al*, 2003). The prognosis of advanced NSCLC patients who receive cisplatin-based chemotherapy is still poor, and the renal and gastrointestinal toxicities caused by cisplatin often limit its clinical use. Therefore, development of different treatment strategies is necessary.

Nedaplatin is a second-generation platinum derivative that has shown equivalent antitumour activity and lower toxicity – less nausea, and lower nephrotoxicity and neurotoxicity – than cisplatin (Kameyama *et al*, 1990; Ota *et al*, 1992). A phase I study demonstrated the maximum tolerated dose (MTD) and the recommended dose (RD) for phase II studies of nedaplatin was 120 and 100 mg m^−2^, respectively, and the dose-limiting toxicity (DLT) was thrombocytopenia (Ota *et al*, 1992). Two independent phase II studies of nedaplatin for NSCLC showed response rates of 14.7 and 20.5%, respectively, and 16.7 and 12.5% with the patients who had received chemotherapy previously (Fukuda *et al*, 1990; Furuse *et al*, 1992a). Based on these promising results, a randomised study of nedaplatin–vindesine *vs* cisplatin–vindesine was conducted for previously untreated NSCLC patients in Japan and indicated that nedaplatin-based chemotherapy yielded similar response rates and overall survival (Furuse *et al*, 1992b). Leucopenia, renal toxicities and gastrointestinal toxicities were more frequent in the cisplatin–vindesine arm, while thrombocytopenia was more frequent in the nedaplatin–vindesine arm.

Gemcitabine, an analogue of deoxycytidine, is a pyrimidine antimetabolite, that shows a reproducible response rates of >20% with a median survival time of 9 months, offering a quality of life benefit in comparison with best supportive care (Abratt *et al*, 1994; Anderson *et al*, 1994; Gatzemeier *et al*, 1996; Anderson *et al*, 2000). The main toxicity of gemcitabine is mild-to-moderate myelosuppression. The combination of gemcitabine and cisplatin showed synergistic effects in preclinical studies because gemcitabine inhibited the repair of DNA damage caused by cisplatin (Bergman *et al*, 1996), and achieved high response rates along with improvements in median survival time in clinical setting (Sandler *et al*, 2000; Schiller *et al*, 2002; Alberola *et al*, 2003).

Recently, carboplatin has attracted attention ahead of nedaplatin because it has similar activity to cisplatin with fewer nonhaematological toxicities. The available data suggest that carboplatin–paclitaxel or carboplatin–gemcitabine should be considered among standard regimen for advanced NSCLC (Kelly *et al*, 2001; Grigorescu *et al*, 2002; Rudd *et al*, 2002; Schiller *et al*, 2002).

It seems that nedaplatin has activity and toxicity profiles similar to those of carboplatin, although no randomised trial has not been done to allow direct comparison (Fukuda *et al*, 1990; Furuse *et al*, 1992a; Ota *et al*, 1992). Moreover, Matsumoto *et al* (2001) demonstrated that the combination of nedaplatin and gemcitabine resulted in enhanced inhibition of tumour growth *in vivo* and the antitumour efficacy of the combination was superior to that of cisplatin–gemcitabine or carboplatin–gemcitabine. Based on the results of a preclinical study, we designed the present phase I study of the efficacy of the combination of nedaplatin and gemcitabine for advanced NSCLC. The purpose of this study was to establish the toxicities and MTD of this combination, to determine the RD for phase II studies, and to observe their antitumour activity.

## PATIENTS AND METHODS

### Patient eligibility

Patients with histologic or cytologic confirmation of locally advanced or metastatic NSCLC who received either no prior chemotherapy or one previous chemotherapy regimen were eligible. The eligibility criteria were as follows; (1) measurable lesions; (2) age ⩽75 years; (3) Eastern Cooperative Oncology Group (ECOG) performance status (PS) 0–1; (4) adequate organ function (a white blood count (WBC) ⩾4000 *μ*l^−1^, a neutrophil count ⩾2000 *μ*l^−1^, a platelet count ⩾100 000 *μ*l^−1^, a haemoglobin count ⩾9.5 g dl^−1^, serum total bilirubin ⩽1.5 mg dl^−1^, serum transaminase ⩽2 × upper normal limits, a serum creatinine ⩽ upper normal limits, blood urea nitrogen (BUN) ⩽25 mg dl^−1^, PaO_2_ ⩾60 mmHg or SpO_2_ ⩾90%]; and (5) normal electrocardiogram (ECG). At least 4 weeks must have passed after the completion of previous therapy and the patients had to have recovered from the toxic effects of previous therapy. The exclusion criteria consisted of pulmonary fibrosis or interstitial pneumonitis with symptoms or apparent abnormalities on chest X-ray, massive pleural effusion or ascites, acute inflammation, pregnancy, lactation, symptomatic brain metastases, active concurrent malignancies, severe drug allergies, severe heart disease, cerebrovascular disease, uncontrollable diabetes mellitus or hypertension, severe infection, active peptic ulcer, ileus, paralysis intestinal, diarrhoea and jaundice. This study was performed at Kinki University School of Medicine and was approved by the Institutional Review Board. Written informed consent was obtained from all patients. This study was conducted in accordance with Declaration of Helsinki.

### Pretreatment and follow-up studies

Prior to entry, a complete history was taken and physical examination including age, height, weight, performance status, histological diagnosis, tumour stage, contents of previous treatment and presence of a complication was performed. The pretreatment laboratory investigations included a complete blood cell count, differential WBC count, platelet count, serum electrolytes, total protein, albumin, total bilirubin, transaminase, alkaline phosphatase, lactate dehydrogenase, BUN, creatinine, creatinine clearance and urinalysis. After the initiation of therapy, a complete blood cell count with a differential WBC count was performed at least twice a week. Blood chemistry profiles and chest X-ray films were obtained weekly. The lesion measurements were performed during at least every second course. Toxicities were evaluated according to the National Cancer Institute Common Toxicity Criteria (NCI-CTC) version 2 and tumour responses were assessed using the Response Evaluation Criteria in Solid Tumors (RECIST) guidelines (Therasse *et al*, 2000). Time to progression was measured from the date of registration to the date of first progression or death from any cause. Survival time was also measured from the date of registration to the date of death or latest follow-up, and was calculated using the Kaplan–Meier method (Kaplan and Meier, 1958).

### Drug administration and dose escalation

The treatment schedule included nedaplatin, diluted with 500 ml of normal saline, given intravenously over 90 min on day 1, and gemcitabine with 100 ml of normal saline, given intravenously over 30 min after the completion of nedaplatin infusion on days 1 and 8, every 3 weeks. All patients were allowed to receive antiemetics with dexamethasone and granisetron, and post-therapy hydration with 1000 ml of normal saline. Granulocyte colony-stimulating factor (G-CSF) prophylaxis was not administered. Doses of gemcitabine on day 8 were given if the WBC count was >2000 *μ*l^−1^ and/or the platelet count was >750 000 *μ*l^−1^, and/or allergic reaction, fever, elevation of transaminase and pneumonitis were less than grade 2, and/or the other nonhaematological toxicities were less than grade 3. The subsequent courses were withheld until the toxic levels returned to those specified in the eligibility criteria. The doses of both drugs were decreased by one dose level if DLTs occurred. In the case of the initial dose level, the doses of nedaplatin and gemcitabine were reduced by 20 and 200 mg m^−2^, respectively.

Dose escalations were performed as listed in Table 1

**Table 1:** Dose-escalation schema

	**Nedaplatin dose**	**Gemcitabine dose**	**No. of patients**
**Dose level**	**(mg m^−2^)**	**(mg m^−2^)**	**(courses)**
1	60	800	3 (8)
2	80	800	3 (10)
3	80	1000	8 (18)
4	100	1000	6 (20)

. Intrapatient dose escalation was not allowed. At least three patients were treated at each dose level, and three additional patients were entered at the same dose level if DLT was observed in one of the first three patients. The MTD was defined as the dose level at which more than two of three patients, or three of six patients experienced DLT. The definition of DLT was as follows: (1) grade 4 leukopenia, (2) grade 4 neutropenia for more than 4 days, (3) thrombocytopenia <20 000 *μ*l^−1^, (4) grade 3 febrile neutropenia, (5) grade 3 nonhaematologic toxicity except for nausea/vomiting, (6) delay of administration of gemcitabine on day 8 over a week for toxicities.

## RESULTS

Between August 2001 and February 2003, 20 patients were enrolled in this study. The total and the median number of courses were 56 and 3 (range 1–6), respectively. The patients’ characteristics are shown in Table 2

**Table 2:** Patients’ characteristics

No. of patients		20
Age, years	Median	63.5
	Range	36–74
Sex	Male/female	17/3
Performance status	0/1	5/15
Histology	Adeno/squamous	13/7
Stage	IIIB/IV	4/16
Prior therapy	None	5
	Surgery	5
	Radiation	6
	Chemotherapy	14
	CDDP-based	3
	CBDCA-based	4
	Nonplatinum	4
	UFT	2
	Gefitinin	1

. The majority of patients had a PS of 1. There were five previously untreated patients (level 3, two patients; level 4, three patients) and 15 (75%) previously treated patients. Of the previously treated patients, five had received prior surgery, five had prior radiotherapy, and 14 had prior chemotherapy. Seven had received platinum-based chemotherapy (cisplatin, three patients; carboplatin, four patients), and four a nonplatinum regimen. Responses to previous chemotherapy included partial response in five patients, stable disease in seven, progressive disease in one, and not evaluable in one. The median interval from previous treatment was 16 weeks (range 4–92.5 weeks). Out of 20 patients, 18 were assessable for toxicity and response. Two patients at level 3 were excluded from the toxicity and response evaluation because they had refused this study after registration.

### Toxicities

The haematological and nonhaematological toxicities observed during the first course are shown in Tables 3

**Table 3:** Haematological toxicity following first course of nedaplatin and gemcitabine

		**WBC grade**					**ANC grade**					**plt grade**					**Hb grade**				
**Dose level**	**No. of patients**	**0**	**1**	**2**	**3**	**4**	**0**	**1**	**2**	**3**	**4**	**0**	**1**	**2**	**3**	**4**	**0**	**1**	**2**	**3**	**4**
1	3	0	2	1	0	0	0	1	2	0	0	0	1	1	1	0	0	2	1	0	0
2	3	1	0	2	0	0	1	0	1	1	0	0	3	0	0	0	0	1	2	0	0
3	6	1	1	2	1	0	2	0	0	3	1	1	2	1	2	0	3	3	0	0	0
4	6	1	0	3	2	0	0	0	3	1	2	0	2	1	3	0	0	3	3	0	0

and 4, respectively. The most frequent toxicities observed in the first cycle were neutropenia and thrombocytopenia (Table 3). One-third of the patients had grade 3 thrombocytopenia, and one patient received a platelet transfusion during the first course. Three patients had grade 4 neutropenia for no longer than 4 days. The nadir for neutropenia and thrombocytopenia occurred on day 15 (median, range 5–18), and on day 15 (median, range 8–18), respectively. Nonhaematological toxicities generally were mild because none of the patients had experienced more than grade 3 in the first course (Table 4

**Table 4:** Nonhaematological toxicity following first course of nedaplatin and gemcitabine

		**Nausea grade**					**Vomiting grade**					**Fatigue grade**					**Transaminase grade**				
**Dose level**	**No. of patients**	**0**	**1**	**2**	**3**	**4**	**0**	**1**	**2**	**3**	**4**	**0**	**1**	**2**	**3**	**4**	**0**	**1**	**2**	**3**	**4**
1	3	3	0	0	0	0	3	0	0	0	0	2	1	0	0	0	3	0	0	0	0
2	3	1	1	1	0	0	3	0	0	0	0	1	2	0	0	0	1	2	0	0	0
3	6	2	3	1	0	0	5	1	0	0	0	4	2	0	0	0	3	1	2	0	0
4	6	2	2	2	0	0	6	0	0	0	0	6	0	0	0	0	1	5	0	0	0
																					
		**Infection grade**					**Fever grade**					**Appetite loss grade**					**Constipation grade**				
**Dose level**	**No. of patients**	**0**	**1**	**2**	**3**	**4**	**0**	**1**	**2**	**3**	**4**	**0**	**1**	**2**	**3**	**4**	**0**	**1**	**2**	**3**	**4**
1	3	3	0	0	0	0	3	0	0	0	0	3	0	0	0	0	3	0	0	0	0
2	3	2	0	1	0	0	2	1	0	0	0	1	2	0	0	0	3	0	0	0	0
3	6	6	0	0	0	0	6	0	0	0	0	2	4	0	0	0	4	2	0	0	0
4	6	4	0	2	0	0	6	0	0	0	0	2	4	0	0	0	4	2	0	0	0

). The major toxicities following all courses are listed in Table 5

**Table 5:** Toxicities following all courses of nedaplatin and gemcitabine (56)

	**Grade**			
	**1**	**2**	**3**	**4**
WBC	13	26	10	0
ANC	15	15	13	3
Hb	24	27	1	0
Plt	22	14	16	0
Nausea	17	4	0	0
Vomiting	6	0	0	0
Appetite loss	21	0	0	0
Fatigue	15	0	0	0
Constipation	6	7	0	0
Transaminase	27	5	0	0
Neuropathy	5	0	0	0
Pneumonitis	0	0	1	0
Fever	1	0	0	0
Infection	0	3	1	0

. Grade 3 thrombocytopenia occurred in 16 out of 56 courses, and three patients received platelet transfusion (one patient at level 1, one at level 3 and one at level 4). However, no patient had haemorrhagic complications. The most frequent nonhaematological toxicities were elevation of transaminase activity, nausea and appetite loss, but all were mild. One previously untreated patient at level 3 experienced grade 3 pneumonitis after the fifth course, probably induced by this treatment, and the patient’s condition improved after the administration of steroid. There was no treatment-related death. One of the 18 patients at level 4 underwent dose reduction after the first course due to neutropenia, and two patients at level 3 did not receive gemcitabine on day 8 because they had neutropenia, thrombocytopenia and high transaminase activity. Delays in the commencement of subsequent courses occurred in 11 courses, and the median length of the delay before starting the subsequent course was 21 days (21–35 days).

### MTD and DLTs

At levels 1 and 2, none of the patients had developed a DLT. Haematological and nonhaematological toxicities were generally mild at these levels, although one patient had grade 3 thrombocytopenia at level 1. At level 3, two of six assessable patients had developed DLTs. Both could not receive their scheduled dose of gemcitabine on day 8 because they had neutropenia, thrombocytopenia and high transaminase activity. At level 4, three of six patients had developed DLTs. One patient received G-CSF for neutropenia, not lasting more than 4 days, which was considered as the DLT. Another patient required a platelet infusion because of thrombocytopenia <20 000 *μ*l^−1^. The third patient could not receive the second course due to the delayed anaemia, also considered as DLT. Therefore, dose level 4, 100 mg m^−2^ nedaplatin with 1000 mg m^−2^ gemcitabine was regarded as the MTD. The recommended dose level for further phase II study was determined to be 80 mg m^−2^ nedaplatin with 1000 mg m^−2^ gemcitabine (dose level 3 in this study).

### Response and survival

There were three partial responses, for an overall response rate of 16.7%. As for squamous cell carcinoma, only one out of seven patients had a partial response. The median progression-free survival time was 5.1 months. The median survival time and 1-year survival rate were 9.1 months and 34.1%, respectively. Out of 15 patients who had received prior treatment, two (13.3%) achieved a partial response, and there was no clear relationship between responses to previous treatment and responses to this regimen. For previously treated patients, the median survival time and 1-year survival rate were 9.2 months and 40.3%, respectively. Among five previously untreated patients, one (20%) achieved a partial response and the median survival time and 1-year survival rate were 12.0 months and 50.0%, respectively.

## DISCUSSION

Many recent randomised clinical trials have shown that the combinations of cisplatin with one of the new agents, such as gemcitabine, taxanes or vinorelbine, is the standard therapy for patients with locally advanced or metastatic NSCLC (Non-Small Cell Lung Cancer Collaborative Group, 1995; Kelly *et al*, 2001; Schiller *et al*, 2002; Fossella *et al*, 2003). As it is known that cisplatin strongly promotes nephrotoxicity, neurotoxicity and gastrointestinal toxicity, second-generation platinum-containing compounds including carboplatin have attracted attention. Based on several randomised trials that have shown that the combination of carboplatin with paclitaxel produces similar response rates and overall survival with a more favourable toxicity profile than the combination of cisplatin with new agents (Kelly *et al*, 2001; Scagliotti *et al*, 2002; Schiller *et al*, 2002), combined therapy of carboplatin and paclitaxel is considered to be a standard therapy. More recently, the combination of carboplatin with gemcitabine has become attractive as a therapy for advanced NSCLC. Some randomised studies have indicated that carboplatin–gemcitabine regimen offers equivalent median survival compared with cisplatin–gemcitabine or mitomycin–vinblastine–cisplatin /mitomycin–ifosfamide–cisplatin (Danson *et al*, 2003; Zatloukal *et al*, 2003), and results in significant improvements in overall survival over those for gemcitabine alone or the older cisplatin-containing regimens (Grigorescu *et al*, 2002; Rudd *et al*, 2002; Sederholm, 2002). However, neutropenia and thrombocytopenia were more common in carboplatin–gemcitabine regimens than others; thrombocytopenia was particularly common.

Like carboplatin, nedaplatin is also a second-generation platinum derivative that appears to have a similar mechanism and toxicity profile to carboplatin, although direct comparison has not been performed. Moreover, *in vivo* study suggested that nedaplatin–gemcitabine resulted in more enhanced inhibition of tumour growth than cisplatin–gemcitabine or carboplatin–gemcitabine. These results prompted us to investigate nedaplatin-based combinations and to conduct this phase I study.

With respect to toxicities, the most frequent toxicities were haematological toxicities, especially neutropenia and thrombocytopenia. Eight of 18 patients (44.4%) developed more than grade 3 neutropenia after the first courses, and after 16 out of 56 (28.6%) courses overall. On the other hand, six out of 16 patients (37.5%) developed grade 3 thrombocytopenia after the first courses, and after 16 out of 56 courses (37.5%) overall. However, patients required platelet transfusions during only three courses. In addition, one previously untreated patient developed drug-related pneumonitis, which improved with the administration of steroid, at level 3 after the fifth course.

Overall, the toxicities of the combination of nedaplatin with gemcitabine were generally mild and this combination chemotherapy is both well tolerated and active against advanced NSCLC.

The overall response rate of 16.7%, the median survival time of 9.1 months, and 1-year survival rate of 34.1% in this study were quite acceptable because most patients had been given prior chemotherapy. As evaluation of antitumour activity was not a primary objective, and our patient population was small and heterogeneous, we are unable to draw definitive conclusions about the activity of this regimen. Currently, it is still controversial whether novel platinum compounds such as carboplatin and nedaplatin could replace cisplatin for the treatment of advanced NSCLC. However, when not only antitumour activity but also palliation are the main goals of treatment, these new platinum compounds might play a useful role because of their favourable toxicity profile. Therefore, nedaplatin–gemcitabine warrants a phase II study, and further evaluation in prospective randomised trials with cisplatin- or carboplatin-based combinations as a first-line chemotherapy for advanced NSCLC in order to investigate whether nedaplatin could replace cisplatin or carboplatin.

In conclusion, the combination of nedaplatin with gemcitabine is well tolerated and active for advanced NSCLC. The MTD and recommended dose level are 100 mg m^−2^ nedaplatin with 1000 mg m^−2^ gemcitabine and 80 mg m^−2^ nedaplatin with 1000 mg m^−2^ gemcitabine, respectively.
